# Pseudo-aneurysm of the anterior tibial artery, a rare cause of ankle swelling following a sports injury

**DOI:** 10.1186/1471-227X-5-9

**Published:** 2005-10-14

**Authors:** Conor D Marron, Damian McKay, Ruth Johnston, Eamon McAteer, WJ Ivan Stirling

**Affiliations:** 1 Department of Surgery, Craigavon Area Hospital, Lurgan Road, Portadown, Northern Ireland

## Abstract

**Background:**

Ankle pain and swelling following sports injuries are common presenting complaints to the accident and emergency department. Frequently these are diagnosed as musculoskeletal injuries, even when no definitive cause is found. Vascular injuries following trauma are uncommon and are an extremely rare cause of ankle swelling and pain. These injuries may however be limb threatening and are important to diagnose early, in order that appropriate treatment can be delivered. We highlight the steps to diagnosis of these injuries, and methods of managing these injuries. It is important for clinicians to be aware of the potential for this injury in patients with seemingly innocuous trauma from sports injuries, who have significant ankle pain and swelling.

**Case presentation:**

A young, professional sportsman presented with a swollen, painful ankle after an innocuous hyper-plantar flexion injury whilst playing football, which was initially diagnosed as a ligamentous injury after no bony injury was revealed on X-Ray. He returned 2 days later with a large ulcer at the lateral malleolus and further investigation by duplex ultrasound and transfemoral arteriogram revealed a Pseudo-Aneurysm of the Anterior Tibial Artery. This was initially managed with percutaneous injection of thrombin, and later open surgery to ligate the feeding vessel. The patient recovered fully and was able to return to recreational sport.

**Conclusion:**

Vascular injuries remain a rare cause of ankle pain and swelling following sports injuries, however it is important to consider these injuries when no definite musculo-skeletal cause is found. Ultrasound duplex and Transfemoral arteriogram are appropriate, sensitive modalities for investigation, and may allow novel treatment to be directed percutaneously. Early diagnosis and intervention are essential for the successful outcome in these patients.

## Background

Ankle pain and swelling are common presenting symptoms to accident and emergency departments following sports injuries[1]. This may be as a result of either blunt, or penetrating trauma to the ankle or surrounding region. In a small proportion of patients there is no definite musculo-skeletal cause determined as a cause of pain and swelling. We report a rare case of an anterior tibial artery pseudo-aneurysm as a cause of ankle swelling following an innocuous hyper-plantar flexion injury. This case illustrates that vascular injury should be considered with an unexplained ankle swelling, in the presence of musculo-skeletal stability, and highlights the steps to accurate diagnosis and management.

## Case presentation

### Case report

A 24 year old male, professional sportsman presented to the accident and emergency department of a district general hospital with ankle pain and swelling following an innocuous hyper-plantar flexion injury to his right ankle by kicking a ball in mid-air, while playing soccer.

**Figure 1:**
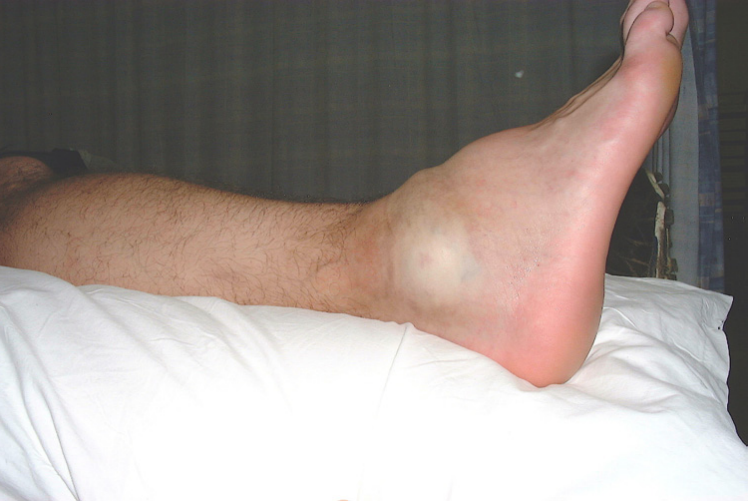
The patient's swollen and discoloured ankle at the time of initial presentation to the Accident and Emergency Department.

The ankle was noted to be swollen with discolouration (Fig. 1). There was localised tenderness at the lateral malleolus. X-Rays of the ankle did not reveal any bony injury. A presumed diagnosis of ligament damage was made and the patient reassured and discharged.

**Figure 2:**
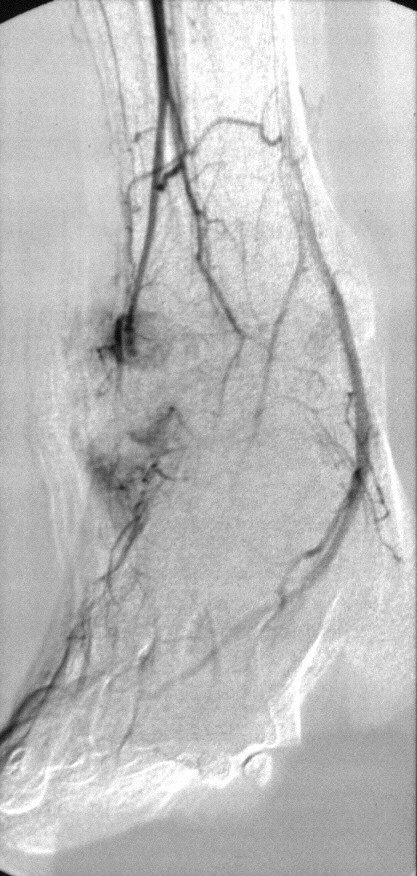
Transfemoral Arteriogram performed demonstrating the Pseudoaneurysm of the Anterior Tibial Artery.

He re-presented to the accident and emergency department 5 days later, having developed an area of skin ulceration 1 cm proximal to the lateral malleolus. Ultrasound Doppler studies were performed, which demonstrated the presence of an anterior tibial artery aneurysm, confirmed by transfemoral arteriogram (Fig 2).

This aneurysm was injected with Thrombin 5000 iu and collagen 200 mg, after written, informed, consent was obtained from the patient, in order to attempt to manage the aneurysm by a percutaneous route using a method well established for the treatment of other pseudoaneurysms. This had a good radiological result with thrombosis of the aneurysm on both duplex and arteriogram.

Progress was monitored via duplex imaging and on day 3 the aneurysm was noted to be partially patent. The patient elected to proceed to surgery with ligation of the anterior tibial artery proximal to the aneurysm. He recovered uneventfully, and at one year follow up has good ankle function allowing him to return to playing recreational soccer, however did not return to professional sport.

## Discussion

Ankle pain and swelling may be a result of either blunt, or penetrating trauma to the ankle or surrounding region.

Vascular injuries following non-penetrating, low energy trauma to the lower limbs are rare. The causes include blunt trauma, laceration from bone fragments, traction injuries, and crush injuries [2,3].

The most common vessel injured is the popliteal artery followed by the superficial femoral, then anterior tibial artery[3]. The proposed mechanism of injury is traction of the vessel against its immobile attachments to long bones[2].

Injuries to the anterior tibial artery are described after interventional procedures such as ankle arthroscopy, however they remain very rare as a result of hyper-plantar flexion or inversion of the ankle, with only a few similar cases reported in literature[2,4,5].

Clinical suspicion of vessel injury is essential in leading to diagnosis, with the indication for further investigation arising from the presence of unexplained swelling, unexplained haemarthrosis, vascular insufficiency, a pulsatile mass, or compartment syndrome. Vascular injury can be limb threatening and delay in diagnosis of these injuries is the commonest cause of complications. Resultant complications vary from severe pain, to ulceration, and possible amputation[2,6].

Ultrasound duplex imaging is accurate in diagnosing injuries to the anterior tibial artery, while transfemoral arteriogram has been the gold standard investigation, as it permits treatment to be commenced [7,8].

Treatment options for pseudo-aneurysm of the anterior tibial artery include coil embolisation, ultrasound guided compression, percutaneous injection of thrombin, and open surgery[8,9]. Surgical options include the ligation of feeding vessel, or primary repair, with or without interposition grafting [5,6]. This case demonstrates the novel use of percutaneous injection of Thrombin to the aneurysm sac, under duplex guidance, as a treatment modality.

Percutaneous treatment using thrombin, although initially successful, does have associated risks, including necrosis and distal embolisation, and there is a potential delay in healing when treatment is not successful. Given these limitations the patient elected for surgery to ligate the feeding vessel, although a further injection of Thrombin was offered and considered.

## Conclusion

This case highlights the potential for vascular injuries with this common mechanism of injury. This should be considered in the differential diagnosis of those presenting with unexplained severe ankle swelling, haemarthrosis, a pulsatile mass, or ulceration following this mechanism of injury, as missed diagnosis can result in significant morbidity and may be limb threatening[6].

Early diagnosis, is essential to the successful management of these injuries[10], and novel percutaneous therapy may represent a viable option for treatment, however this requires further evaluation.

## Competing interests

The author(s) declare that they have no competing interests.

## Authors' contributions

CM was involved in the conception of the idea for the manuscript, the diagnosis and operative management of the patient, and preparation of the manuscript.

DmcK was involved in the operative and outpatient management of the patient, and has reviewed, and amended the manuscript for submission.

RJ was responsible for ward management of the patient, literature review, critical reading of the manuscript and some re-writing of the manuscript for submission.

EmcA was responsible for the radiological investigation and management of the patient, as well as critically reviewing the manuscript and amending it for submission.

WJIS was responsible for the diagnosis, operative and outpatient management of the patient as the consultant in charge of the case. He was involved in the conception of the idea for the report, reviewing the manuscript and amending for submission.

All authors have read and approved the manuscript for submission.

## Pre-publication history

The pre-publication history for this paper can be accessed here:


